# Cannabidiol or ketamine for preventing the impact of adolescent early drug initiation on voluntary ethanol consumption in adulthood

**DOI:** 10.3389/fphar.2024.1448170

**Published:** 2024-08-27

**Authors:** Carles Colom-Rocha, Cristian Bis-Humbert, M. Julia García-Fuster

**Affiliations:** ^1^ University Research Institute of Health Sciences, University of the Balearic Islands, Palma, Spain; ^2^ Health Research Institute of the Balearic Islands (IdISBa), Palma, Spain; ^3^ Department of Medicine, University of the Balearic Islands, Palma, Spain

**Keywords:** addiction risk factors, adolescence, alcohol use disorder, sex differences, therapeutical options, rodent models

## Abstract

**Background:**

Few studies have previously evaluated the long-term impact of initiating the combined use of alcohol and cocaine early-in-life during adolescence. Our preclinical study characterized changes in affective-like behavior and/or voluntary ethanol consumption emerging later on in adulthood induced by a prior adolescent drug exposure, as well as tested therapeutical interventions (i.e., cannabidiol or ketamine) to prevent the observed effects.

**Methods:**

We performed three independent studies with male and female Sprague-Dawley rats, treated in adolescence (postnatal days, PND 29–38) with non-contingent paradigms of ethanol, cocaine, their combination or vehicle. Later on, adult rats were (1) scored for their affective-like state (forced-swim, elevated-plus maze, novelty-suppressed feeding, sucrose preference), (2) allowed to freely drink ethanol for 6 weeks (two-bottle choice), or (3) treated with cannabidiol or ketamine before given access to ethanol in adulthood.

**Results:**

No signs of increased negative affect were observed in adulthood following the adolescent treatments. However, adolescent ethanol exposure was a risk-factor for later developing an increased voluntary ethanol consumption in adulthood, both for male and female rats. This risk was similar when ethanol was combined with adolescent cocaine exposure, since cocaine alone showed no effects on later ethanol intake. Finally, rats exposed to adolescent ethanol and pretreated in adulthood with cannabidiol (and/or ketamine, but just for females) reduced their ethanol voluntary consumption.

**Conclusion:**

Our data provided two therapeutical options capable of preventing the impact of an early drug initiation during adolescence by decreasing voluntary ethanol consumption in adult rats

## Highlights

Adolescent ethanol exposure is a risk-factor for later voluntary ethanol consumption.
This increased vulnerability is observed both for male and female rats.
Adding cocaine exposure in adolescence does not increase the observed risk.
Cannabidiol (and/or ketamine, but just for females) reduced the observed risk.

## Background

Besides our individual biological genetic predisposition, several other factors might be responsible for a higher risk of developing substance use disorder later in life, especially early drug initiation during adolescence (Morales et al., 2020; Nawi et al., 2021). Interestingly, adolescence is a period of critical brain development that appears to be highly conserved across species in terms of its neurobehavioral and physiological features. Similar to the stages observed in humans, early (10–13 years), middle (14–17 years), and late adolescence/young adulthood [18–21 years (Christie and Viner, 2005; Backes and Bonnie, 2019)], in rodents, adolescence could be divided into early (postnatal day, PND 21–34), mid (PND 34-46) and late adolescence [PND 46-59 (Spear, 2004)]. Therefore, the use of rodents provides a great preclinical model (García-Fuster, 2021; Nieto et al., 2021) with face and construct validity (Spear, 2004) in which to study particular windows of adolescent vulnerability to psychopathology and therapeutic strategies (Adriani and Laviola, 2004).

In this context, our research group, among others [reviewed by Steinfeld and Torregrossa (2023)], aimed at characterizing adolescent windows of vulnerability during which the use of illicit drugs could be more harmful, both behaviorally and neurochemically (García-Cabrerizo et al., 2015; García-Cabrerizo and García-Fuster, 2016; García-Cabrerizo and García-Fuster, 2019; García-Fuster et al., 2017; Parsegian et al., 2022). In particular, a regimen of adolescent cocaine exposure known to induce psychomotor sensitization was more harmful during PND 33-39 than other earlier (PND 26-32) or later (PND 40-46) windows of adolescence (García-Cabrerizo et al., 2015), showing signs of increased negative affect (García-Cabrerizo and García-Fuster, 2019) and addictive-like behaviors (García-Fuster et al., 2017; Parsegian et al., 2022) when rats were re-exposed to cocaine in adulthood, as well as changes in neurochemical markers of neurotoxicity (García-Cabrerizo et al., 2015; García-Cabrerizo and García-Fuster, 2016; García-Fuster et al., 2017; Parsegian et al., 2022). Although all of these studies were done in male rats, we also recently reported that adolescent cocaine induced persistent negative affect in adult female rats (Bis-Humbert and García-Fuster, 2021). Therefore, this window and pattern of cocaine exposure during mid-adolescence is of high vulnerability to the long-term effects emerging in adulthood and caused by an early drug initiation.

Interestingly, cocaine is rarely consumed alone, since it is frequently combined with alcohol; out of the individuals who reported heavy alcohol use in the past month, 5% also consumed cocaine (Graziani et al., 2014). Both the European Monitoring Centre for Drugs and Drug Addiction (EMCDDA) as well as the National Center for Drug Abuse Statistics (NCDAS) in US, yearly report that alcohol is by far the most commonly consumed substance among teens and young adults. This data proves an earlier initiation and a higher use for alcohol, as compared to cocaine, and therefore suggests that alcohol initiation will be generally sooner, and that the combined use of both drugs would parallel the time at which cocaine’s use is started. The many prior studies evaluating the effects of adolescent alcohol consumption suggested that the behavioral irregularities (i.e., elevations in anxiety, disinhibition, impulsivity and risk-taking, decreased cognitive flexibility) and neural consequences of adolescent alcohol use may persist into adulthood [reviewed by Spear (2016); Spear (2018); Lees et al. (2020)], including an increased ethanol drinking in adulthood (Strong et al., 2010; Sherrill et al., 2011). However, studies evaluating the combined impact of alcohol and cocaine have been centered mainly in the cardiotoxic effects mediated by cocaethylene (the active metabolite produced by both drugs), so there is a need for novel data on the long-term impact of initiating alcohol alone and/or combined with cocaine during adolescence on later alcohol use disorder rates in adulthood.

Against this background, we characterized the changes induced by an even earlier start of adolescent ethanol exposure at PND 28, as compared to the one previously depicted for cocaine initiation (vulnerability window starting on PND 33), as well as their combined impact on the effects emerging in adulthood, and while including both sexes in the study (Towers et al., 2023; Becker and Koob, 2016), since most of the data summarized above was done in male rodents. Particularly, we assessed affective- (i.e., behavioral despair and/or stress-coping mechanisms, anxiety-related behaviors, anhedonic-like response) and/or addictive-like behaviors [i.e., voluntary consumption: 20% ethanol two-bottle choice paradigm (Colom-Rocha et al., 2023)]. Then, we selected the worst outcome induced by prior adolescent drug exposure, to test whether two therapeutical interventions (cannabidiol or ketamine) would prevent the increased ethanol consumption emerging in adulthood (Yardley and Ray, 2017). These options were chosen based on previous preclinical and/or clinical data suggesting an amelioration of ethanol-motivated behaviors both by cannabidiol (Viudez-Martínez et al., 2018; Viudez-Martínez et al., 2020; Nona et al., 2019; Maccioni et al., 2022; Tringali et al., 2023; Gasparyan et al., 2023) or ketamine [reviews by Worrell and Gould (2021); Goldfine et al. (2023); Kelson et al. (2023); Krystal et al. (2024)], although most of these studies were tested in either pups from pregnant rodents exposed to drugs during gestation, and/or in rodents that received drugs in adolescence and/or adulthood, but were tested at the same age-window of study, and therefore, data evaluating the possible preventive effects of these options following an early adolescent drug experience are missing. A pre-print version of this manuscript has been uploaded to Research Square (https://doi.org/10.21203/rs.3.rs-3943360/v1).

## Methods

### Animals

**FIGURE 1:**
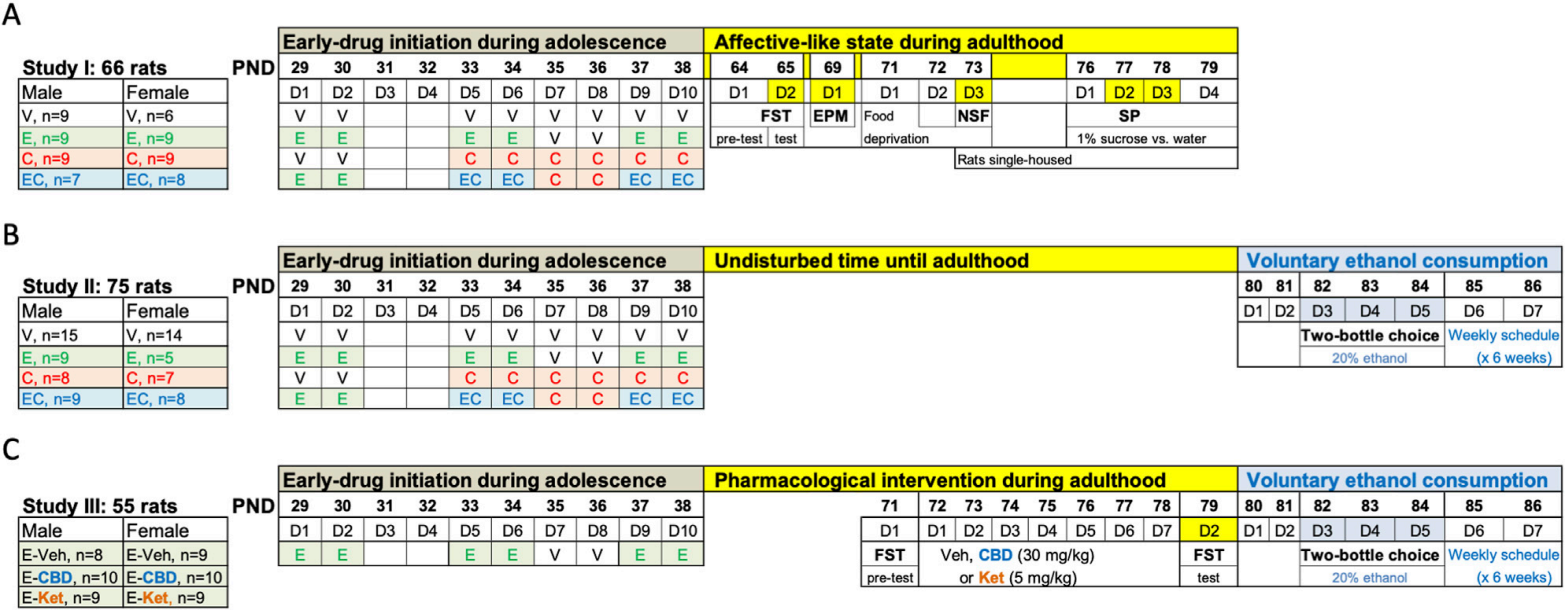
Experimental timeline. **(A)** Affective-like state during adulthood following adolescent drug exposure. **(B)** Evaluating voluntary ethanol consumption in adulthood following adolescent drug exposure. **(C)** Pharmacological intervention during adulthood before voluntary ethanol access. **(A–C)** Adolescent drug treatments were done at the indicated times: vehicle (V: 0.9% NaCl, 1 mL/kg/day, i. p.), ethanol (E: 2 g/kg, i. p.), cocaine (C: 15 mg/kg/day, i. p.), and/or their combination (EC: ethanol + cocaine as described). EPM, elevated plus maze; FST, forced swim test; NSF, novelty-suppressed feeding test; PND, post-natal day; SP, sucrose preference.

A total of 196 Sprague-Dawley rats (102 males, 94 females) were bred in the animal facility at the University of the Balearic Islands. After weaning (PND 21), groups of allocated rats were used in three independent studies (Figure 1). Unless otherwise specified, rats were housed (groups of 2–4) in standard cages following a 12 h light/dark schedule (lights on at 8:00 AM) in a climate-controlled room (22°C, 70% humidity) and with limitless access to a standard diet and water. Procedures were performed during the light-period and complied with ARRIVE Guidelines (Percie du Sert et al., 2020), EU Directive 2010/63/EU, and Spanish Royal Decree 53/2013, requiring prior approval by the Local Bioethical Committee (CEEA 148-09-20) and Regional Government (2021/01/AEXP). All efforts were made to minimize the number of rats used, the number of procedures and their suffering. To avoid unnecessary stress in females, the specific stages of the estrous cycle were not monitored, since cyclicity of females was not part of our research question (Beltz et al., 2019) and females seem as variable as males due to hormonal periodicity (Becker et al., 2016; Kaluve et al., 2022) (reinforced by the observed individual variability for males and females in this study).

### Pharmacological treatments during adolescence

The exposure paradigm followed was based on the combination of two well-known prior paradigms for each drug separately. On one end, a psychomotor sensitizing regimen of cocaine exposure has been extensively characterized in adolescence by our research group, even the impact of the drugs at different windows during this developmental time-period (i.e., early, middle vs late adolescence) (García-Cabrerizo et al., 2015; García-Cabrerizo and García-Fuster, 2016; García-Cabrerizo and García-Fuster, 2019; García-Fuster et al., 2017; Parsegian et al., 2022). We selected the window starting at PND 33 that we have characterized in more detail in terms of inducing long-term changes in affective- and addictive-like behaviors (García-Cabrerizo et al., 2015; García-Cabrerizo and García-Fuster, 2016; García-Cabrerizo and García-Fuster, 2019; García-Fuster et al., 2017; Parsegian et al., 2022), which therefore defined the timing and cocaine regimen followed in the present experiment. On the other end, since ethanol exposure needed to start sooner than cocaine, to mimic the pattern of consumption in adolescents, ethanol was initiated on PND 29 and was administered in an intermittent fashion following a binge design. In particular, allocated adolescent male and female rats were treated with ethanol (2 g/kg, i. p; 3 rounds of 2 days at 48-h intervals; PND 29-30, PND 33-34 and PND 37–38 [Pascual et al., 2007; Pascual et al., 2017; Crabbe et al., 2011)], cocaine [15 mg/kg/day, i. p., 6 days from PND 33-38, as previously characterized (García-Cabrerizo et al., 2015; García-Cabrerizo and García-Fuster, 2016; García-Cabrerizo and García-Fuster, 2019; García-Fuster et al., 2017; Parsegian et al., 2022)], or their combination (ethanol + cocaine as described above). Also, ethanol was administered via i. p. Injections as opposed to oral gavage so it could be combined and given concomitantly with cocaine as detailed in Figure 1. Vehicle (0.9% NaCl) was administered at the indicated days in the control group, but also at the days no ethanol or cocaine was programmed in comparison to the ethanol + cocaine group (Figure 1). The same experimenter was involved in all pharmacological administrations and/or procedures. We relied on a non-contingent regime of drug exposure in adolescence, so we could account for the same dosing in each experimental group as opposed to the individual values we would have obtained for each rat if a contingent regime was followed.

### Affective-like state in adulthood following adolescent drug exposure

Rats from Study I were left undisturbed until adulthood (PND 64), when they were scored across time through a battery of tests following standard protocols (Bodnoff et al., 1988; Slattery et al., 2007; Slattery and Cryan, 2012) that measure different affective-like dimensions: forced-swim (FST: PND 64-65), elevated plus maze (EPM: PND 69), novelty-suppressed feeding (NSF: PND 73), and sucrose preference through the two-bottle choice test (SP: PND 77-78) (Figure 1A).

The FST, which is regularly performed in our group (García-Fuster et al., 2012; García-Fuster and García-Sevilla, 2016; Bis-Humbert et al., 2021; Ledesma-Corvi et al., 2022), consisted of a pre-test session, when rats were placed in individual tanks (41 cm high × 32 cm diameter, 25 cm depth) filled with water (25°C ± 1°C) during 15 min (D1: PND 64) to learn there is no escape, followed by a 5-min test session (D2: PND 65), when the behavioral response was videotaped (Figure 1A). Clean water tanks were used for each rat. Videos were blindly analyzed by two independent experimenters (Behavioral Tracker, CA, United States): increased immobility rates as a measure of behavioral despair vs swimming or climbing a measure of escaping behaviors. The number of feces were quantified at the end of the test session as a measurement of distress (correlated with a higher number of feces).

The EPM was later performed in a black Plexiglas maze with four elevated arms (50 cm from ground × 50 cm long × 10 cm wide), two open and two closed arms with 40 cm high walls. Each rat (PND 69) was placed for 5-min in the central square (10 cm × 10 cm) facing a closed arm (García-Fuster et al., 2012) and allowed to freely explore under red-lighting. The maze was cleaned with 70% ethanol between animals. Individual sessions were recorded with a Logitech HD webcam c270 and were analyzed through a computerized tracking system (SMART, v.3.0.06; Panlab Harvard Apparatus^®^, Barcelona, Spain) that provided several measurements: latency to open arm (s), number of open vs closed arms entries, time spent in open vs closed arms (s). Besides latency to open arms (s), results were expressed as percent open arm entries (%) and percent open arm time (%). Percent open time was calculated by dividing open time by open + closed time, thus discounting the time spent in the center compartment of the apparatus.

Then, and in an attempt to acquire the food motivation required for the NSF test, rats were food-deprived for 48 h from PND 71-73 (see Figure 1A), following similar prior procedures (Bis-Humbert et al., 2021; Ledesma-Corvi et al., 2022). On test day (PND 73), each rat was placed at one of the corners facing the wall of the square open-field arena (60 cm × 60 cm, 40 cm high walls), and was allowed to freely explore the arena for 5 min under housing illumination conditions with three food pellets in the center (Bis-Humbert et al., 2021; Ledesma-Corvi et al., 2022). Sessions were videotaped to then analyze feeding time (s), total distance traveled (cm) and latency to food (s). The arena was cleaned in between animals with 70% ethanol to avoid potential behavioral interferences caused by individual odors.

Finally, rats were exposed to the SP test. To do so and prior to testing, rats were single-housed (PND 73) to obtain individual drinking values. Rats were trained to drink from two water bottles placed on each side of the housing cage for 24 h (PND 76), then, for the next 2 days, they were given access to one bottle containing 1% sucrose and the other one containing water (PND 77-78; Figure 1A) (García-Cabrerizo and García-Fuster, 2019; Jiménez-Romero et al., 2020; Bis-Humbert et al., 2020; Bis-Humbert et al., 2021; Ledesma-Corvi et al., 2022). Bottles were placed in alternate cage positions to prevent specific-side preferences. On PND 79, rats were presented with two water bottles for 24 h, to control for bias for either bottle and/or side of the cage. Bottles were daily weighted to calculate sucrose preference (%) and intake (g/kg).

### Voluntary ethanol consumption in adulthood following adolescent drug exposure

Rats from Studies II and III were allowed to voluntarily drink ethanol (20%) for a total of 6 weeks by a two-bottle choice test, starting on PND 80 (Figures 1B, C), and thus were exposed to the same experimental conditions. This provided four experimental groups/sex that only differed in adolescent drug exposure. The difference in sample sizes is related to the need of splitting the study in two independent waves while including enough controls in both of them. The design followed an intermittent access to 20% ethanol described to exert a steady consumption sustained over time, with higher overall doses for females (Colom-Rocha et al., 2023). In particular, this procedure allowed, on a weekly basis and for a total of 6 weeks, unlimited voluntary access to ethanol (20% ethanol vs. water) for 72 consecutive hours (3 days from Tuesdays to Thursdays, D3-D5, Figure 1) followed by a 4-day period with no ethanol access (access to two water-bottles: D6-D7, D1-D2) every week (i.e., a total of 18 ethanol sessions of 24 h). Ethanol bottles were located in alternate positions (right of left side) to account for potential side preferences. Bottles were weighed every morning during the 3 days of weekly ethanol access. Results are expressed (average consumption during all sessions) in terms of ethanol preference (%), water or ethanol volume consumed (ml/24 h), and ethanol dose (g/kg/24 h). Ethanol preference was calculated as the amount of ethanol consumed (mL) divided by total fluid intake (sum of both bottles in ml) and multiplied by 100 (% values).

### Pharmacological intervention before drug re-exposure in adulthood

Given that adolescent ethanol exposure increased voluntary drug consumption in adulthood (as described in Study II), in an attempt to reduce the number of animals used (3Rs), all rats from Study III were exposed to ethanol in adolescence. No vehicle group was included in adolescence since that comparison was already evaluated in Study II. Plus, our goal was to ascertain whether the pharmacological intervention would decrease the increased voluntary ethanol consumption observed in adulthood driven by the prior adolescent experience. The idea was to pharmacologically treat rats in adulthood right before drug re-exposure (i.e., allowing voluntary ethanol access), with either cannabidiol [30 mg/kg, i. p (Bis-Humbert et al., 2020; Gálvez-Melero et al., 2023)], ketamine [5 mg/kg, i. p (Ledesma-Corvi et al., 2023)], or vehicle for seven consecutive days (PND 72-78; Figure 1C), to ascertain their prevention on the impact of adolescent ethanol on voluntary consumption. Also, drugs were scored for their potential antidepressant-like responses in the FST (pre-test: PND 71; 5-min test: PND 79) (Bis-Humbert et al., 2020; Gálvez-Melero et al., 2023; Ledesma-Corvi et al., 2023). Since the FST affected all groups in a similar fashion, any decreases observed in ethanol preference and/or consumption would prove a therapeutical option for the tested drugs (cannabidiol or ketamine vs vehicle-treated rats in adulthood).

### Data analyses and statistics

All data analyses and graph plotting were done with GraphPad Prism, Version 10 (GraphPad Software, United States) following guidelines in experimental pharmacology for displaying data and statistical methods (Michel et al., 2020). Results are reported as mean values ±standard error of the mean (SEM); individual symbols are shown for each rat within bar-graphs. Assumptions for normality of data distribution and homogeneity of variance were met. Supplementary Table S1 includes two-way ANOVAs (independent variables: Sex, Treatment) of all data evaluated. Sex effects are also reported in graphs (Dalla et al., 2024). Since expected sex-differences were initially hypothesized in affective-like responses (Jiménez-Romero et al., 2020; Ledesma-Corvi et al., 2023) and ethanol consumption (Colom-Rocha et al., 2023), but also in the potential therapeutical response induced by cannabidiol (Gálvez-Melero et al., 2023) or ketamine (Ledesma-Corvi et al., 2023) (see main effects of Sex when performing two-way ANOVAs in Supplementary Table S1), male and female rats were analyzed separately through one-way ANOVAs. Multiple comparisons tests were performed for *post hoc* comparisons when appropriate (Dunnett’s). Level of significance: *p* ≤ 0.05. Data supporting the present findings will be available upon reasonable request to the corresponding author.

## Results

### No changes in the affective-like state of adult rats following adolescent drug exposure

**FIGURE 2:**
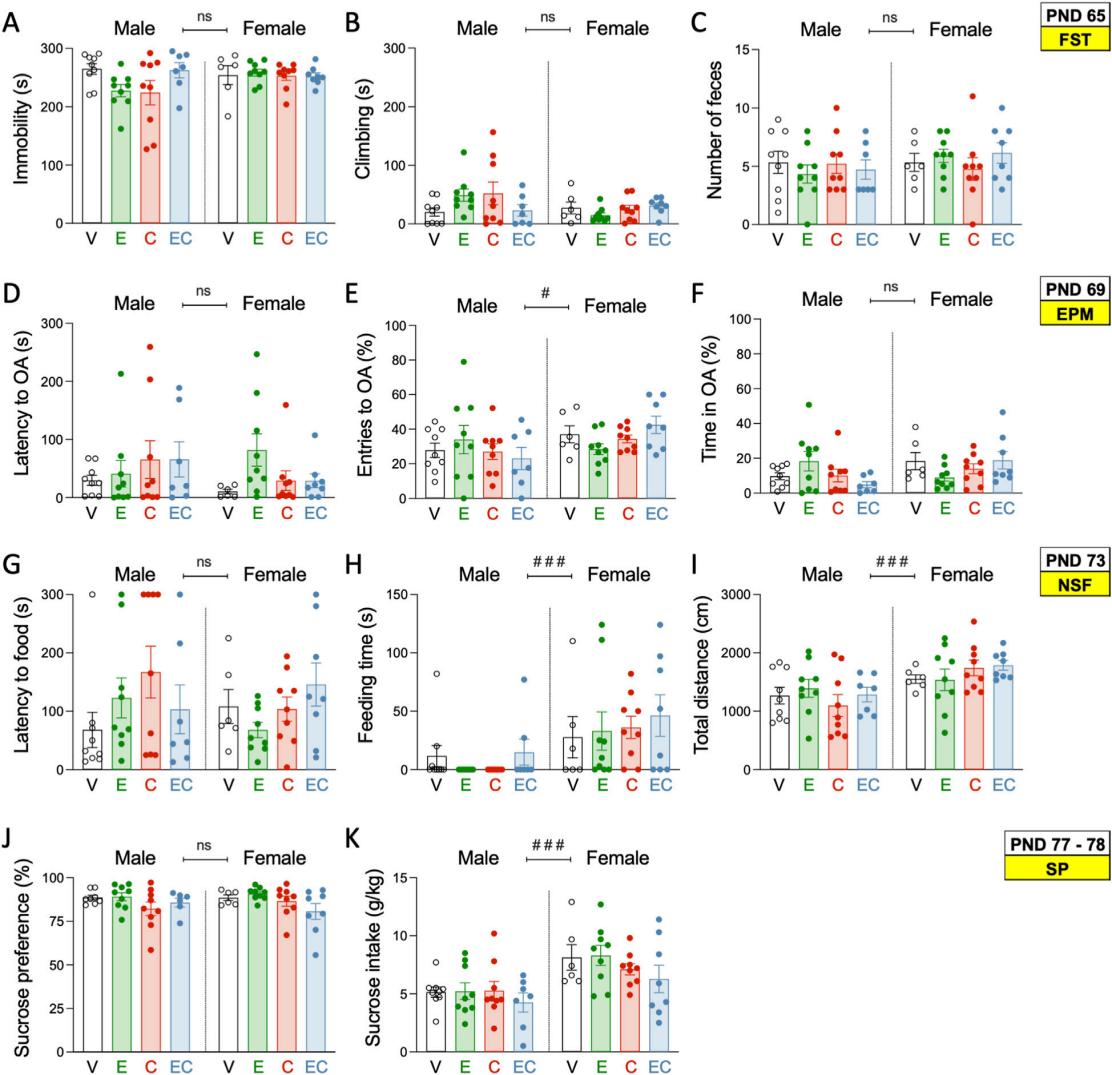
Affective-like state during adulthood following adolescent drug exposure. **(A)** Time spent immobile (s) or **(B)** climbing (s), and **(C)** number of feces in the forced swim test (FST) on PND 65. **(D)** Latency to open arms (OA) (s), **(E)** OA entries, and **(F)** time spent in OA (s) in the elevated plus maze test (EPM) on PND 69. **(G)** Latency to food (s), **(H)** feeding time (s), and **(I)** distance travelled (cm) in the novelty-suppressed feeding test (NSF) on PND 73. **(J)** Sucrose preference (%) and **(K)** sucrose intake (g/kg) in the sucrose preference test (SP) on PND 77–78. **(A–K)** Each set of data represents the mean ± SEM of the corresponding measurement at the indicated PND of study. Individual values are shown for each rat (symbols). Groups of treatment: vehicle-male (n = 9); ethanol-male (n = 9); cocaine-male (n = 9); ethanol + cocaine-male (n = 7); vehicle-female (n = 6); ethanol-female (n = 9); cocaine-female (n = 9); ethanol + cocaine-female (n = 8). One-way ANOVAs (independent variable: Treatment) were performed for each sex separately, and are detailed in Supplementary Table S1. The table also includes two-way ANOVAs analyses with Sex and Treatment as independent variables (Effect of Sex: ^###^ *p* < 0.001 when comparing female vs male rats). V: vehicle; E: ethanol; C: cocaine; EC: combination.

An earlier adolescent drug treatment (ethanol, cocaine or their combination) did not induce changes in the affective-like state of male or female rats during adulthood, and as measured in FST (Figures 2A–C), EPM (Figures 2D–F), NSF (Figures 2G–I) and SP (Figures 2J, K; Supplementary Table S1 for further statistical details). Moreover, additional analyses were performed to estimate individual behavioral phenotypes for each rat (Z-score: affective-like state for all tests combined). This was done following prior literature suggesting that combining normalized values from many different behavioral measurements of different, but complementary, behavioral tests could be more precise than just analyzing individual items, to characterize a “depressive-like syndrome” in rodents (von Mücke-Heim et al., 2023). However, no significant changes in affective-like Z-scores were observed among treatment groups (Supplementary Figure S1).

As expected, and previously hypothesized, some sex differences were observed in the tests performed. For example, in the NSF, female rats spent considerably longer times feeding (mean of 36 s vs. 7 s for males, ###*p* < 0.001; Figure 2H), while also travelled longer distances (mean of 1,654 cm vs. 1,259 cm for males, ###*p* < 0.001; Figure 2I). Moreover, females showed higher sucrose intake (+2.5 ± 0.6%, g/kg vs. males, ###*p* < 0.001; Figure 2K). These sex-differences suggested decreased anxiogenic- and improved hedonic-like responses in females, as compared to males, independently of the adolescent treatment.

### Increased voluntary ethanol consumption in adult rats following adolescent ethanol exposure: lack of cocaine effects

**FIGURE 3:**
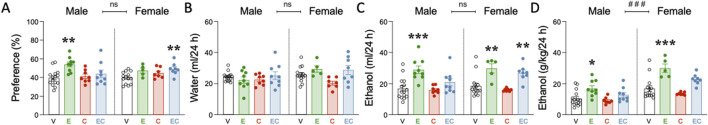
Voluntary ethanol consumption in adulthood following adolescent drug exposure. **(A)** ethanol preference (%), **(B)** water intake (ml/24 h), **(C)** ethanol intake (ml/24 h), and **(D)** ethanol dose (g/kg/24 h). **(A–D)** Columns represent mean ± SEM of the preference for ethanol (expressed as a % value), water or ethanol intake (ml/24 h) and ethanol dose consumed (g/kg/24 h) in the two-bottle choice test (20% ethanol vs water choice). Individual values are shown for each rat (symbols). Groups of treatment: vehicle-male (n = 15); ethanol-male (n = 9); cocaine-male (n = 8); ethanol + cocaine-male (n = 9); vehicle-female (n = 14); ethanol-female (n = 5); cocaine-female (n = 7); ethanol + cocaine-female (n = 8). One-way ANOVAs (independent variable: Treatment) were performed for each sex separately, and are detailed in Supplementary Table S1. Dunnett’s *post hoc* analyses: *p < 0.05, **p < 0.01, and ***p < 0.001 vs same-sex vehicle-treated rats. The table also includes two-way ANOVAs analyses with Sex and Treatment as independent variables (Effect of Sex: ^###^ *p* < 0.001 when comparing female vs male rats). V: vehicle; E: ethanol; C: cocaine; EC: combination.

A prior adolescent drug treatment induced long-term changes in voluntary ethanol consumption for male and female adult rats (Figure 3), as observed by significant ANOVAs (Supplementary Table S1) for ethanol preference, ethanol intake (ml/24 h), and ethanol dose (g/kg/24 h). *Post-hoc* analysis revealed that adult male rats treated with ethanol in adolescence showed increased preference (+14.4 ± 3.8%, **p = 0.017; Figure 3A), a higher volume of ethanol consumed (+12.1 ± 2.7 mL/24 h, ***p < 0.001; Figure 3C), and a higher overall dose (+6.5 ± 1.9 g/kg/24 h, **p = 0.005; Figure 3D) than adolescent vehicle-treated rats, without changing the amount of water consumed (Figure 3C). Interestingly, adolescent cocaine had no effect on ethanol voluntary consumption in adulthood, as was also the case when combining ethanol with cocaine (Figures 3A–D).

On the other hand, adult female rats treated with adolescent ethanol also showed a higher volume of ethanol consumed (+11.2 ± 3.1 mL/24 h, **p = 0.003; Figure 3C), and a higher overall dose (+13.1 ± 2.8 g/kg/24 h, ***p < 0.001; Figure 3D) than vehicle-treated female rats (Figure 3C). While adolescent cocaine lacked significant effects over ethanol voluntary consumption, the combined exposure of ethanol and cocaine showed increased ethanol preference (+9.2 ± 2.8%, **p = 0.064; Figure 3A) and consumption (+8.4 ± 2.6 mL/24 h, **p = 0.009; Figure 3C) in adult female rats.

Again, and as previously described (Colom-Rocha et al., 2023), some sex differences were observed at the level of the dose of ethanol consumed, with female rats showing an overall higher dose (+8.5 ± 1.2 g/kg/24 h, ###*p* < 0.001) than males (Figure 3D; Supplementary Table S1). However, no sex differences were observed for ethanol preference, and/or ethanol or water volume consumed (Figures 3A–C).

### Cannabidiol or ketamine diminished voluntary ethanol consumption in adult rats following adolescent ethanol exposure

**FIGURE 4:**
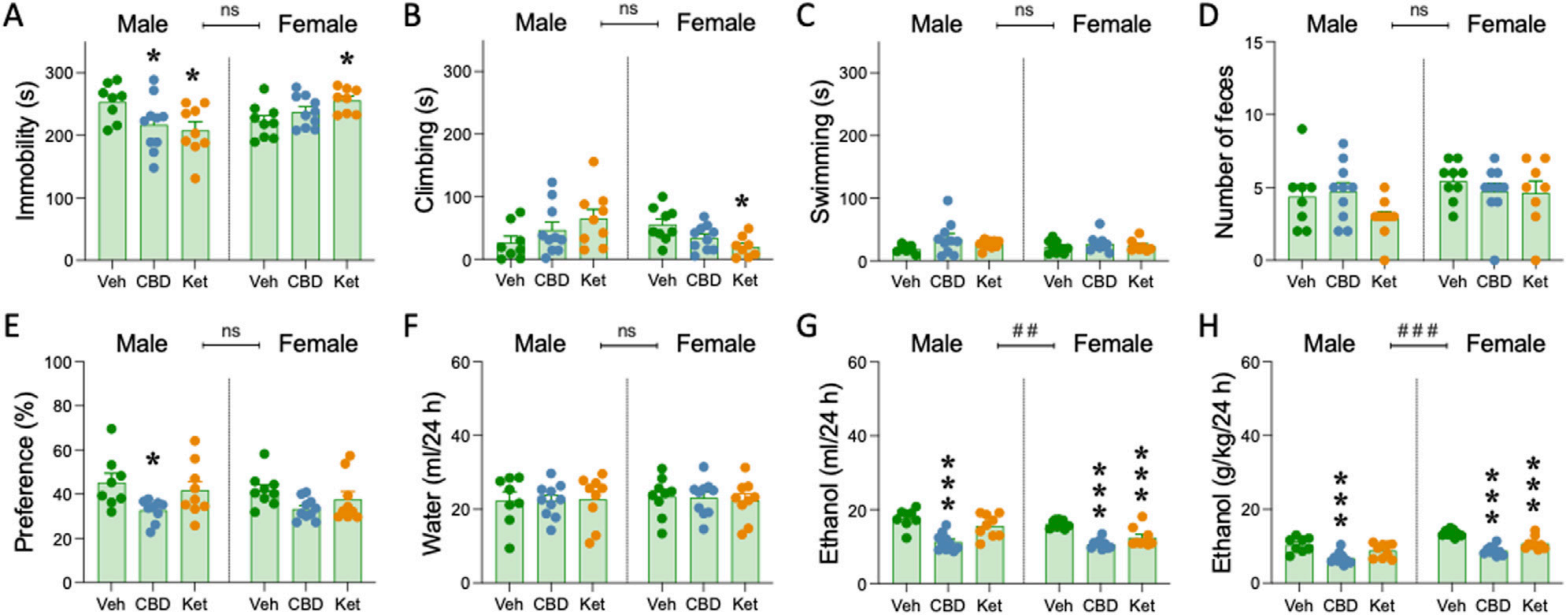
Pharmacological intervention before ethanol re-exposure in adulthood. **(A)** Time spent immobile (s), **(B)** climbing (s) or **(C)** swimming (s), and **(D)** number of feces in the forced swim test FST in adult rats following adolescent ethanol exposure. **(E)** ethanol preference (%), **(F)** water intake (ml/24 h), **(G)** ethanol intake (ml/24 h), and **(H)** ethanol dose (g/kg/24 h). **(A–F)** Columns represent mean ± SEM of the different scores for each test. Individual values are shown for each rat (symbols). Groups of treatment: vehicle-male (n = 8); cannabidiol-male (n = 10); ketamine-male (n = 9); vehicle-female (n = 9); cannabidiol-female (n = 10); ketamine-female (n = 9). One-way ANOVAs (independent variable: Treatment) were performed for each sex separately, and are detailed in Supplementary Table S1. Dunnett’s *post hoc* analyses: *p < 0.05 and ***p < 0.001 vs same-sex vehicle-treated rats. The table also includes two-way ANOVAs analyses with Sex and Treatment as independent variables (Effect of Sex: ^##^ *p* < 0.01 and ^###^ *p* < 0.001 when comparing female vs male rats). V: vehicle; CBD: cannabidiol; Ket: ketamine. Colom-Rocha, Bis-Humbert and García-Fuster.

Cannabidiol or ketamine induced antidepressant-like responses in the FST in male rats, as observed by a decreased immobility (−36.4 ± 15.4 s, *p = 0.040 and −45.5 ± 15.7 s, *p = 0.011 respectively; Figure 4A; Supplementary Table S1) when compared to vehicle-treated rats. However, cannabidiol was not efficacious in female rats, and ketamine even induced an increase in immobility (+33.5 ± 11.9 s, *p = 0.017; Figure 4A; Supplementary Table S1). These results paired with opposite changes in climbing behavior (Figure 4B), while swimming (Figure 4C) or the number of feces were not altered by any of the treatments tested (Figure 4D; Supplementary Table S1).

As for the effects of the therapeutical intervention after adolescent ethanol exposure, but right before ethanol re-exposure in adulthood, adult male rats pretreated with cannabidiol showed a drop in preference (−12.6% ± 4.9%, *p = 0.033; Figure 4E), a lower volume of ethanol consumed (−6.4 ± 1.3 mL/24 h, ***p < 0.001; Figure 4G), and a lower overall dose (−3.6 ± 0.9 g/kg/24 h, ***p < 0.001; Figure 4H) than rats pretreated with vehicle, and without changing the amount of water consumed (Figure 4F; Supplementary Table S1).

Interestingly, in female rats, both cannabidiol and ketamine showed signs of efficacy on later improving ethanol consumption in adulthood. In particular, both pretreatments were capable of lowering the volume of ethanol (−5.4 ± 0.8 mL/24 h, ***p < 0.001 and −3.7 ± 0.8 mL/24 h, ***p < 0.001, respectively; Figure 4G), as well as the overall dose consumed (−4.7 ± 0.6 g/kg/24 h, ***p < 0.001 and −2.7 ± 0.6 g/kg/24 h, ***p < 0.001, respectively; Figure 4H), vs. rats pretreated with vehicle, and without changing water consumption (Figure 4F; Supplementary Table S1).

In line with prior results (Figure 3), some sex differences were reported, both at the level of ethanol consumption and overall dose (Supplementary Table S1), with female rats showing overall higher rates (volume consumed: +1.8 ± 0.6 mL/24 h, ##*p* = 0.006; dose: +2.4 ± 0.4 g/kg/24 h, ###*p <* 0.001) than males (Figures 4G, H; Supplementary Table S1). No sex differences were observed for ethanol preference or water volume consumed (Figures 4E, F).

## Discussion

This study examined the long-term changes in affective-like responses and in ethanol voluntary consumption in adult male and female rats previously exposed to ethanol, cocaine or their combination during vulnerable windows of adolescence. The main results showed that exposing rats to these drugs in adolescence did not induce signs of increased negative affect during adulthood, although this conclusion is limited to the doses, regimens and methods of administration followed. However, when rats were also allowed to voluntarily consume ethanol in adulthood, some differences emerged depending on the drug they previously received in adolescence. In particular, adolescent ethanol exposure was a risk-factor for later developing an increased voluntary ethanol consumption in adulthood, both in male and female rats. This risk was similar when ethanol was combined with cocaine exposure in adolescence, since adolescent cocaine exposure alone did not affect ethanol intake in adulthood. Finally, rats exposed to ethanol in adolescence and pretreated in adulthood with either cannabidiol (and/or ketamine but just for females) reduced ethanol voluntary consumption. Overall, our data provided two therapeutical options capable of attenuating voluntary ethanol consumption rates in adulthood caused by a prior adolescent drug exposure.

This study initially hypothesized that the combined exposure of ethanol and cocaine in adolescence would show increased sings of negative affect in adult male and female rats. In this context, a recent review compared the behavioral phenotypes that emerge following different ethanol exposure models, and concluded that the complex outcomes from these studies highlighted the difficulties of assessing negative affective behaviors in rodent models designed for the study of alcohol use disorder (Bloch et al., 2022). In our case, the behavioral phenotyping was done through the sequential screening in different tests that try to capture different aspects of the symptomatology observed in humans, such as behavioral despair (García-Fuster et al., 2012) and/or stress-coping strategies (Molendijk and de Kloet, 2015; Commons et al., 2017) through FST, anxiety-like responses in EPM (García-Fuster et al., 2012) and NSF (Bis-Humbert et al., 2021; Ledesma-Corvi et al., 2022), and hedonic-like responses in SP (Jiménez-Romero et al., 2020). However, we found no evidence of a deteriorated affective-like state induced by any of the drugs administered in adolescence (ethanol, cocaine or their combination) in rats of both sexes. Note that adolescent drug exposure was administered by an experimenter in a non-contingent way so each experimental group received the same dose in terms of later being able to better understand the impact of a prior fixed drug exposure on ethanol voluntary intake in adulthood. Although this type of regimen did not inform about the motivation for the drug during adolescence, and might have impacted the lack of negative affect observed during drug abstinence, it is worth mentioning that this set of data aligned with our prior results suggesting that adolescent drug exposure alone may not be sufficient to induce negative affect in adulthood, since adult drug re-exposure was needed to observe a negative impact on behavior from an earlier adolescent exposure (García-Cabrerizo and García-Fuster, 2019; Bis-Humbert and García-Fuster, 2021; Bis-Humbert et al., 2021). Also, in this line of thought, adding stress could also be a possible factor that can reveal the phenotype, suggesting that adolescent early exposure to drugs increased susceptibility or vulnerability to later consumption. This idea reinforces the notion that one way to prevent substance use disorder later on relies on avoiding drug consumption early in life. Moreover, in line with the previously detected basal sex-differences for these particular tests of study (Hernández-Hernández et al., 2023; Ledesma-Corvi et al., 2023), the data reported a differential impact by sex in some tests, with a greater general exploratory-like behavior for females as observed in NSF, combined with a higher hedonic-like response in SP.

The next study evaluated the impact of the adolescent treatment on ethanol consumption later on in adulthood. Although no changes were observed when measuring affective-like responses, when exposing rats to a voluntary access to ethanol in adulthood [20% ethanol for three consecutive days per week, during 6 weeks; characterized in Colom-Rocha et al. (2023)], some differences in consumption emerged depending on the drug they previously received in adolescence. In particular, and in line with prior data (Strong et al., 2010; Sherrill et al., 2011) [reviewed by Spear (2016); Spear (2018); Lees et al. (2020)], adolescent ethanol exposure was a clear risk-factor for later developing an increased voluntary ethanol consumption in adulthood, both in male and female rats. In particular, previous adolescent ethanol exposure increased ethanol preference and voluntarily consumption in adulthood, being this effect of equal magnitude for both sexes. However, when correcting ethanol intake by weight to calculate the dose consumed per day (g/kg/24 h), female rats were exposed to higher doses than their male counterparts, in line with prior recent examples showing sex-specific drinking patterns in adult rodents (Colom-Rocha et al., 2023; Foo et al., 2023; McElroy et al., 2023). Moreover, the risk observed by ethanol combined with cocaine in adolescence was of a similar magnitude, or even a bit reduced, to the one induced just by ethanol, since adolescent cocaine exposure alone did not affect ethanol consumption in adulthood. Remarkably, the literature is full of data showing how adolescent ethanol exposure alters the rewarding effects of cocaine in adulthood (Hutchison and Riley, 2012; Mateos-García et al., 2015; Ledesma et al., 2017; Esteve-Arenys et al., 2017; Cantacorps et al., 2020), however, studies evaluating how adolescent cocaine impact later adult ethanol consumption are lacking. One example described the acute effects of cocaine stimulating ethanol intake in a two-bottle choice paradigm, but was done in adult male Sprague-Dawley rats (Colvin et al., 2022), Therefore, since adolescent cocaine exposure did not stimulate ethanol intake in adulthood, the effects observed by ethanol and cocaine combined in adolescence over the rate of voluntary ethanol consumption in adulthood might be driven exclusively by prior ethanol exposure in adolescence. These results reported that despite the fact that adolescent drug exposure did not induce any clear signs of negative affect, when adult rats were presented with a voluntary drug experience, the prior adolescent experience had clear consequences on the emerging addictive-like behaviors observed for both sexes. Although these negative effects were not observed for all drugs (lack of impact by adolescent cocaine), adolescent ethanol exposure increased the rates of ethanol consumption in adulthood. Interestingly, adolescent alcohol use was also proven a risk factor for adult alcohol and drug dependence in a clinical studies with twins (Grant et al., 2006). Therefore, since an early exposure to ethanol during adolescence induced long-term consequences for substance use disorders later on in adulthood, preventing an early-age initiation is postulated to be of vital importance to avoid future problems related to substance use disorders. However, for cases when adolescent drug exposure is already initiated, having access to therapeutical options that could prevent a later drug consumption when ethanol renders available in adulthood are of great relevance.

In this line of thought, our third study tested two therapeutical interventions (cannabidiol, ketamine) for preventing the increased ethanol consumption in adult rats following a prior adolescent ethanol exposure. Note that this experiment was done in the absence of ethanol-naive controls (i.e., all rats received ethanol in adolescence), since our prior experiment already characterized the long-term effects of ethanol vs vehicle exposure in adolescence, and the present goal was to ascertain whether the tested therapeutical options could reduce the voluntary drinking observed in adulthood and caused by a prior adolescent experience. In particular, the main results showed that rats exposed to ethanol in adolescence and pretreated in adulthood with cannabidiol (and/or ketamine but just for females) reduced ethanol voluntary consumption in adulthood. Interestingly, the rates of ethanol preference and/or consumption in adult male and female rats were similar to the ones observed in our prior study for the adolescent ethanol group, demonstrating that independent studies replicated and were therefore reliable. Moreover, both cannabidiol and ketamine showed antidepressant-like responses in FST in male rats exposed to ethanol in adolescence, in line with prior results in naïve rats (Bis-Humbert et al., 2020; Ledesma-Corvi et al., 2023), while rendered inefficacious or even deleterious for female rats (Ledesma-Corvi et al., 2022; Gálvez-Melero et al., 2023; Ledesma-Corvi et al., 2023). Prior data have already proven sex-differences in antidepressant-like responses, with clear drops in efficacy for female subjects (Kokras et al., 2011; LeGates et al., 2019). Clearly, the antidepressant-like response observed in FST in rats treated with adolescent ethanol did not parallel the response that these therapeutical options induced in terms of ethanol voluntary consumption rates (as detailed below), hence suggesting a broader therapeutical potential, other than their antidepressant-like action, both for cannabidiol and ketamine in adult rats of both sexes.

In accordance with this, for example, both male and female rats pretreated with cannabidiol showed decreased ethanol intake in adulthood as observed by a drop in preference (although only significant in male rats), a lower volume of ethanol consumed, and a lower overall dose than rats pretreated with vehicle. These results aligned with previous preclinical data suggesting an improvement of ethanol-motivated behaviors by cannabidiol [reviewed by Nona et al. (2019)]; see also (Viudez-Martínez et al., 2018; Viudez-Martínez et al., 2020; Maccioni et al., 2022; Tringali et al., 2023; Gasparyan et al., 2023)], including the cognitive deficits and neuroinflammation induced by early ethanol exposure (García-Baos et al., 2021). Particularly, prior studies have proven cannabidiol efficacy in pups from pregnant rodents exposed to drugs during gestation, directly in adolescence (immediate effects), and/or in rodents that received drugs in adulthood. For example, cannabidiol (30 mg/kg/day, i. p., for up to 4–6 weeks) repaired the behavioral and brain disturbances in offspring exposed to an animal model of fetal alcohol spectrum disorder (Gasparyan et al., 2023). When administered during adolescence, cannabidiol (40 mg/kg) before each drinking session reduced ethanol consumption and preference in male rats that underwent the intermittent 20% ethanol two-bottle choice paradigm (Tringali et al., 2023). Moreover, during adulthood, the administration of cannabidiol in male mice reduced the reinforcing properties, motivation and relapse for ethanol (Viudez-Martínez et al., 2018). Later on, a follow-up study from the same group described sex differences in the effects of cannabidiol on ethanol binge drinking in adult mice, proving, similarly to the results obtained here, that although female mice exhibited higher ethanol intake during each drinking in the dark session, cannabidiol reduced ethanol consumption for both sexes; these effects were observed after an acute (90 mg/kg; highest dose tested) and/or chronic administration [although with different dose-dependent efficacy for males and females; males: 30, 60 and 90 mg/kg; females: only with 90 mg/kg (Viudez-Martínez et al., 2020)]. In another study in adult male rats, cannabidiol (≥12.5 mg/kg) markedly reduced lever responding for ethanol and amount of self-administered ethanol in selectively bred Sardinian ethanol-preferring male rats, a validated animal model of excessive ethanol consumption (Maccioni et al., 2022). Against this pool of published data, our study is quite original and novel in that it proposed a different approach, since it evaluated the beneficial effects of cannabidiol on preventing the impact of early drug initiation during adolescence on voluntary ethanol consumption in adult male and female rats. Taken together, these findings, in conjunction with the prior published data, suggested that cannabidiol may be a great candidate for preventing the development of alcohol-use disorders, independently of when drug exposure was initiated (i.e., during prenatal period, adolescence, and/or adulthood), and independently of sex, if previously adjusting the specific sex-related conditions required for efficacy (i.e., dose, length of treatment, animal species, etc.).

Finally, the other therapeutical option tested, at the conditions used, was only capable of showing signs of improvement in female rats. Mainly, ketamine showed signs of efficacy by decreasing ethanol consumption in adulthood, but only in female rats exposed to ethanol in adolescence. In particular, it lowered the volume as well as the overall dose of ethanol consumed, as compared to rats pretreated with vehicle. In line with our results, some prior studies also showed a greater sensitivity to the ketamine treatment in females, although ketamine significantly reduced both ethanol intake and preference in a time- and dose-dependent manner in ethanol preferring adult rats of both sexes, while it did so at higher doses [effective doses: 7.5 mg/kg, and 10 mg/kg (Rezvani et al., 2017)] than the ones used in the present study. Another experiment also reported a better therapeutical response for females by reporting that ketamine reduced binge-like drinking behavior exclusively in female rats exposed to a drinking in the dark model and when ketamine was given prior to ethanol exposure (Crowley et al., 2019). Contrarily, other studies found a greater sensitivity for ketamine in male rodents, for example, while ketamine decreased ethanol consumption in male rats that self-administered high levels of ethanol, it increased ethanol consumption in female rats that showed low levels of ethanol self-administration (Strong et al., 2019). Also, adolescent exposure to ketamine significantly decreased preference for ethanol consumption in males, with a smaller reduction of ethanol consumption in females (Franco et al., 2020). In general, prior results with ketamine at the preclinical and clinical level reported some beneficial effects on ameliorating ethanol-motivated behaviors for both sexes [see recent reviews by Worrell and Gould (2021); Goldfine et al. (2023); Kelson et al. (2023); Krystal et al. (2024)], although with some differences in between sexes that should be further studied since they will likely play an important role in the future development of therapeutical options for alcohol use disorders. Overall, the use of ketamine for the treatment and/or prevention of alcohol use disorder is promising, with several ongoing clinical trials, but with still the need to further complete its efficacy validation and safety profile before recommending its broader clinical use [reviewed by Goldfine et al. (2023)].

## Perspectives and significance

These results provided two therapeutical options for preventing the effects of an early drug initiation during adolescence on ethanol voluntary consumption rates later on in adulthood. Thus, these results may be of relevance in view of possible future studies testing cannabidiol or ketamine in patients affected by alcohol use disorder, whose consumption started early in life during adolescence, and when sex-related differences might be affecting the treatment outcome.

## Data Availability

The original contributions presented in the study are included in the article/Supplementary Material, further inquiries can be directed to the corresponding author.
