# Supplementary material for: Cannabidiol or ketamine for preventing the impact of adolescent early drug initiation on voluntary ethanol consumption in adulthood
Source: Front Pharmacol. 2024 Aug 27;15:1448170. doi: 10.3389/fphar.2024.1448170 (PMC11384591; doi:10.3389/fphar.2024.1448170)

Supplementary Materials

Table. S1. This table represents the one-way ANOVAs analyses for each sex analyzed separately, as well as the two-way ANOVAs analyses, both including F (DFn,DFd) and *p* values, for data represented in Fig. 2-4. Green-shadow boxes represent statistically significant comparisons.

		One-way ANOVA: F (DFn, DFd), *p* value		Two-way ANOVA: F (DFn, DFd), *p* value		
**Figure 2**		**Treatment - Male rats**	**Treatment - Female rats**	**Sex**	**Treatment**	**Sex x Treatment**
**FST**	**A.** Immobility (s)	F(3,30)=2.30; *p* =0.097	F(3,28)=0.11; p=0.851	F(1,58)=1.29; *p* =0.261	F(3,58)=1.50; *p* =0.235	F(3,58)=1.83; *p* =0.152
	**B.** Climbing (s)	F(3,30)=1.69; *p* =0.190	F(3,28)=1.26; p=0.309	F(1,58)=2.46; *p* =0.122	F(3,58)=0.75; *p* =0.529	F(3,58)=2.24; *p* =0.093
	Swimming (s)	F(3,30)=1.97; *p* =0.141	F(3,28)=0.90; p=0.454	F(1,58)=0.45; *p* =0.504	F(3,58)=2.39; *p* =0.078	F(3,58)=0.18; *p* =0.912
	**C.** Feces (number)	F(3,30)=0.31; *p*= 0.820	F(3,28)=0.58; *p*= 0.634	F(1,58)=1.10; *p* =0.298	F(3,58)=0.11; *p* =0.957	F(3,58)=0.73; *p* =0.538
**EPM**	**D.** Latency to OA (s)	F(3,30)=0.52; *p* =0.671	F(3,28)=2.53; p=0.077	F(1,58)=0.64; *p* =0.427	F(3,58)=1.11; *p* =0.351	F(3,58)=1.40; *p* =0.252
	**E.** Entries to OA (%)	F(3,30)=0.55; *p* =0.655	F(3,28)=2.5; *p* =0.079	F(1,58)=4.33; *p* =0.042	F(3,58)=0.08; *p* =0.972	F(3,58)=2.03; *p* =0.120
	**F.** Time in OA (%)	F(3,30)=2.11; *p* =0.120	F(3,28)=1.62; *p* =0.207	F(1,58)=2.63; *p* =0.110	F(3,58)=0.17; *p* =0.916	F(3,58)=3.65; *p* =0.018
**NSF**	**G.** Latency to food (s)	F(3,30)=1.25; *p* =0.309	F(3,28)=1.67; *p* =0.197	F(1,58)=0.15; *p* =0.703	F(3,58)=0.96; *p* =0.418	F(3,58)=1.56; *p* =0.210
	**H.** Feeding time (s)	F(3,30)=1.35; *p* =0.278	F(3,28)=0.24; *p* =0.870	F(1,58)=12.46; *p*< 0.001	F(3,58)=0.58; *p* =0.632	F(3,58)=0.28; *p* =0.841
	**I.** Total distance (cm)	F(3,30)=0.64; *p* =0.597	F(3,28)=0.86; *p* =0.472	F(1,58)=13.79; *p*< 0.001	F(3,58)=0.28; *p* =0.841	F(3,58)=1.19; *p* =0.323
**SP**	**J.** Preference (%)	F(3,30)=1.11; *p* =0.361	F(3,28)=2.19; *p* =0.110	F(1,58)=1.29; *p* =0.260	F(3,58)=2.68; *p* =0.055	F(3,58)=0.05; *p* =0.985
	**K.** Sucrose intake (g/kg)	F(3,30)=0.41; *p* =0.749	F(3,28)=1.09; *p* =0.372	F(1,58)=18.59; *p* <0.001	F(3,58)=1.34; *p* =0.272	F(3,58)=0.32; *p* =0.808
**Figure 3**		**Treatment - Male rats**	**Treatment - Female rats**	**Sex**	**Treatment**	**Sex x Treatment**
**TBC**	**A.** Preference (%)	F(3,37)=5.12; *p*= 0.004	F(3,30)=4.40; *p*= 0.011	F(1,67)=0.09; *p* =0.771	F(3,67)=6.52; *p* <0.001	F(3,67)=1.58; *p* =0.203
	**B.** Water (ml/24 h)	F(3,37)=0.70; *p*= 0.558	F(3,30)=3.37; *p*= 0.026	F(1,67)=3.55; *p* =0.064	F(3,67)=3.11; *p*= 0.032	F(3,67)=1.855; *p* =0.146
	**C.** Ethanol (ml/24 h)	F(3,37)=8.07; *p*< 0.001	F(3,30)=11.05; *p*< 0.001	F(1,67)=1.98; *p* =0.164	F(3,67)=15.16; *p* <0.001	F(3,67)=0.75; *p* =0.524
	**D.** Ethanol (g/kg/24 h)	F(3,37)=5.27; *p*= 0.004	F(3,30)=11.05; *p*< 0.001	F(1,67)=49.66; *p* <0.001	F(3,67)=16.95; *p* <0.001	F(3,67)=2.26; *p* =0.090
**Figure 4**		**Treatment - Male rats**	**Treatment - Female rats**	**Sex**	**Treatment**	**Sex x Treatment**
**FST**	**A.** Immobility (s)	F(2,24)=3.24; *p*= 0.050	F(2,24)=4.00; *p*= 0.032	F(1,48)=1.94; *p* =0.170	F(2,48)=0.49; *p* =0.618	F(2,48)=6.533; *p* =0.003
	**B.** Climbing (s)	F(2,24)=2.034; *p*= 0.154	F(2,24)=5.78; *p*= 0.009	F(1,48)=1.21; *p* =0.278	F(2,48)=0.02; *p* =0.984	F(2,48)=5.81; *p* =0.006
	**C.** Swimming (s)	F(2,24)=2.09; *p*= 0.146	F(2,24)=0.55; *p*= 0.582	F(1,48)=0.39; *p* =0.536	F(2,48)=2.65; *p* =0.081	F(2,48)=0.70; *p* =0.501
	**D.** Feces (number)	F(2,24)=2.37; *p*= 0.115	F(2,24)=0.52; *p*= 0.601	F(1,48)=3.29; *p* =0.008	F(2,48)=1.87; *p* =0.174	F(2,48)=1.00; *p* =0.375
**TBC**	**A.** Preference (%)	F(2,23)=3.53; *p*= 0.046	F(2,25)=2.80; *p*= 0.080	F(1,48)=0.85; *p* =0.360	F(2,48)=6.32; *p* =0.004	F(2,48)=0.37; *p* =0.692
	**B.** Water (ml/24 h)	F(2,24)=0.01; *p*= 0.995	F(2,25)=0.10; *p*= 0.903	F(1,49)=0.10; *p* =0.748	F(2,49)=0.03; *p* =0.974	F(2,49)=0.07; *p* =0.935
	**C.** Ethanol (ml/24 h)	F(2,24)=13.68; *p*< 0.001	F(2,25)=22.74; *p*< 0.001	F(1,49)=8.40; *p* =0.006	F(2,49)=31.37; *p* <0.001	F(2,49)=1.68; *p* =0.197
	**D.** Ethanol (g/kg/24 h)	F(2,24)=8.82; *p*= 0.001	F(2,25)=28.97; *p*< 0.001	F(1,49)=29.78; *p* <0.001	F(2,49)=30.68; *p*< 0.001	F(2,49)=0.68; *p* =0.677

Fig. S1. Affective-like state in adulthood following prolonged drug withdrawal. An affective-like state Z-score was calculated with all the individual measurements recorded from all behavioral tests (FST, EPM, NSF, SP; see von Mücke-Heim et al., 2023). The results showed a lack of significant effect of Treatment ($F_{3,58}$=0.02, *p*=0.997), but a significant effect of Sex ($F_{1,58}$=12.77, ###*p*<0.001), in line with some of the differences already reported in Fig. 2, suggesting females showed overall better affective-like responses than males (+0.25 ± 0.1-fold-increase in affective-like-Z-score females as compared to males). This result supported a lack of a negative affective-like state induced by the adolescent drug treatment in adulthood during prolonged forced-withdrawal.

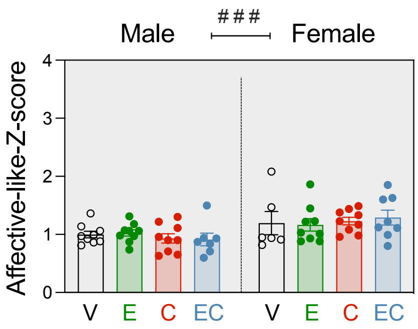

Supplement: Supplementary file 1 [file DataSheet1.PDF]
